# Associations between life-course-persistent antisocial behaviour and brain structure in a population-representative longitudinal birth cohort

**DOI:** 10.1016/S2215-0366(20)30002-X

**Published:** 2020-03

**Authors:** Christina O Carlisi, Terrie E Moffitt, Annchen R Knodt, Honalee Harrington, David Ireland, Tracy R Melzer, Richie Poulton, Sandhya Ramrakha, Avshalom Caspi, Ahmad R Hariri, Essi Viding

**Affiliations:** a Division of Psychology and Language Sciences, University College London, London, UK; b Department of Psychology and Neuroscience, Duke University, Durham, NC, USA; c Department of Psychiatry and Behavioral Sciences, Duke University, Durham, NC, USA; d Center for Genomic and Computational Biology, Duke University, Durham, NC, USA; e Laboratory of NeuroGenetics, Duke University, Durham, NC, USA; f Social, Genetic, and Developmental Psychiatry Research Centre, Institute of Psychiatry, Psychology, and Neuroscience, King's College London, London, UK; g Dunedin Multidisciplinary Health and Development Research Unit, Department of Psychology, University of Otago, Dunedin, New Zealand; h Department of Medicine, University of Otago, Dunedin, New Zealand; i New Zealand Brain Research Institute, Christchurch, New Zealand; j Brain Research New Zealand—Rangahau Roro Aotearo Centre of Research Excellence, Dunedin, New Zealand

## Abstract

**Background:**

Studies with behavioural and neuropsychological tests have supported the developmental taxonomy theory of antisocial behaviour, which specifies abnormal brain development as a fundamental aspect of life-course-persistent antisocial behaviour, but no study has characterised features of brain structure associated with life-course-persistent versus adolescence-limited trajectories, as defined by prospective data. We aimed to determine whether life-course-persistent antisocial behaviour is associated with neurocognitive abnormalities by testing the hypothesis that it is also associated with brain structure abnormalities.

**Methods:**

We used structural MRI data collected at 45 years of age from participants in the Dunedin Study, a population-representative longitudinal birth cohort of 1037 individuals born between April 1, 1972, and March 31, 1973, in Dunedin, New Zealand, who were resident in the province and who participated in the first assessment at 3 years of age. Participants underwent MRI, and mean global cortical surface area and cortical thickness were extracted for each participant. Participants had been previously subtyped as exhibiting life-course-persistent, adolescence-limited, or no history of persistent antisocial behaviour (ie, a low trajectory group) based on informant-reported and self-reported conduct problems from the ages of 7 years to 26 years. Study personnel who processed the MRI images were masked to antisocial group membership. We used linear estimated ordinary least squares regressions to compare each antisocial trajectory group (life-course persistent and adolescence limited) with the low trajectory group to examine whether antisocial behaviour was related to abnormalities in mean global surface area and mean cortical thickness. Next, we used parcel-wise linear regressions to identify antisocial trajectory group differences in surface area and cortical thickness. All results were controlled for sex and false discovery rate corrected.

**Findings:**

Data from 672 participants were analysed, and 80 (12%) were classified as having life-course-persistent antisocial behaviour, 151 (23%) as having adolescence-limited antisocial behaviour, and 441 (66%) as having low antisocial behaviour. Individuals on the life-course-persistent trajectory had a smaller mean surface area (standardised β=–0·18 [95% CI −0·24 to −0·11]; p<0·0001) and lower mean cortical thickness (standardised β=–0·10 [95% CI −0·19 to −0·02]; p=0·020) than did those in the low group. Compared with the low group, the life-course-persistent group had reduced surface area in 282 of 360 anatomically defined parcels and thinner cortex in 11 of 360 parcels encompassing circumscribed frontal and temporal regions associated with executive function, affect regulation, and motivation. Widespread differences in brain surface morphometry were not observed for the adolescence-limited group compared with either non-antisocial behaviour or life-course-persistent groups.

**Interpretation:**

These analyses provide initial evidence that differences in brain surface morphometry are associated with life-course-persistent, but not adolescence-limited, antisocial behaviour. As such, the analyses are consistent with the developmental taxonomy theory of antisocial behaviour and highlight the importance of using prospective longitudinal data to define different patterns of antisocial behaviour development.

**Funding:**

US National Institute on Aging, Health Research Council of New Zealand, New Zealand Ministry of Business, Innovation and Employment, UK Medical Research Council, Avielle Foundation, and Wellcome Trust.

## Introduction

Children and adolescents exhibiting persistent antisocial behaviour are often diagnosed with conduct disorder. These children are at an increased risk for incarceration and poor physical and mental health later in life.^1^ Longitudinal cohort studies have demonstrated marked individual differences in the age of onset and long-term stability of antisocial behaviour. Some individuals display early-onset, life-course-persistent antisocial behaviour, whereas for others antisocial behaviour arises in adolescence but is limited to the pre-adult period (adolescence-limited antisocial behaviour).^2^ Studies in population-representative samples have estimated the prevalence of life-course-persistent antisocial behaviour to be less than 10%, whereas prevalence of adolescence-limited antisocial behaviour is greater than 25%.^3^ The developmental taxonomy theory distinguishing these two forms of antisocial behaviour^2^ has been influential in guiding early-years prevention and juvenile justice policies, as well as in clinical practice, as the taxonomy led to DSM-classified subtypes of childhood-onset versus adolescence-onset conduct disorder.^4^

**Research in context**

**Evidence before this study**

The developmental taxonomy theory of antisocial behaviour outlines two prototypes of antisocial behaviour: life-course persistent and adolescence limited. We searched PubMed and Google Scholar for research articles published between Aug 1, 2018, and April 30, 2019, using the terms “antisocial behavio(u)r”, “conduct problems/disorder”, and “developmental taxonomy”, and we reviewed publications reporting structural brain measures. English language publications were included. Longitudinal studies have documented abnormal neuropsychological development in individuals with life-course-persistent but not adolescence-limited antisocial behaviour. However, no studies have reported on differences in structural brain integrity between these two groups defined by data-driven models.

**Added value of this study**

This study addresses limitations of existing research by examining a population-representative longitudinal birth cohort and by using statistically defined groups of individuals with life-course-persistent or adolescence-limited antisocial behaviour. Providing support for the developmental taxonomy theory, individuals on the life-course-persistent antisocial trajectory had thinner cortex and smaller surface area in brain regions associated with executive function, motivation, and affect, including the ventromedial prefrontal and orbitofrontal cortices, superior temporal gyrus and posterior cingulate cortex, than did individuals who were not antisocial, and these differences were more broadly distributed across the brain than previously reported in cross-sectional studies of antisocial individuals. Moreover, the adolescence-limited group had thinner cortex in the right middle and inferior temporal lobe, regions that have not been associated with antisocial behaviour previously.

**Implications of all the available evidence**

The developmental taxonomy theory has affected juvenile justice policy across the globe as well as clinical diagnosis and treatment. Our study supports the developmental taxonomy theory by showing that individuals on the life-course-persistent but not adolescence-limited trajectory show robust, widespread structural brain differences compared with individuals without antisocial behaviour.

Large, well defined cohort studies indicate that individuals on the life-course-persistent trajectory display difficult childhood temperament and antisocial behaviour early in life.^1^, ^5^, ^6^ Longitudinal studies have found extensive evidence for early neuropsychological impairments in individuals on the life-course-persistent trajectory, particularly in the domains of verbal intelligence quotient (IQ), executive, and memory functions.^7^ Collectively, this evidence suggests that individuals on the life-course-persistent trajectory have neuropsychological vulnerabilities, which, alongside external environmental factors, deny them the opportunity to gain prosocial life skills that promote desistance from antisocial behaviour, and are likely to be linked to underlying neurobiological differences.^8^ However, direct support for the abnormal brain development hypothesis in life-course-persistent antisocial behaviour from in-vivo neuroimaging measures has been scarce.

Adolescence-limited antisocial behaviour emerges alongside puberty and reflects socially normative peer processes, including gaining and asserting independence from adults.^9^ Adolescence-limited antisocial behaviour is thought to develop as a result of adolescents navigating these socially difficult years when a maturity gap indicates mismatch between biological maturation and access to mature responsibilities and relationships. Consequently, many adolescents emulate a delinquent lifestyle to assert autonomy from parents. However, because pre-adolescent neuropsychological development and family environment factors were otherwise normal in these individuals, they desist from antisocial behaviour upon naturally ageing into adult roles.^9^ Effects of this maturity gap have been repeatedly investigated,^10^ and the majority of prospective longitudinal data suggest that neuropsychological impairment is less strongly associated with adolescence-limited than with life-course-persistent antisocial behaviour.^9^ Limited cross-sectional studies show that children with adolescence-onset antisocial behaviour might exhibit atypical emotional and motivational processing that is not generally captured in standard neuropsychological batteries.^11^ However, these studies have not involved large, representative samples defined by prospective data. Thus, a rigorous test of the developmental taxonomy theory regarding differential brain development is needed.

The original formulation of the taxonomy specified abnormal brain development as a fundamental aspect of the aetiology of life-course-persistent but not of adolescence-limited antisocial behaviour,^2^ suggesting that neuroimaging investigations of brain structure would help to develop an understanding of the neurobiological mechanisms delineating these groups. However, neuroimaging investigations of differences in brain structure between antisocial behaviour subtypes as defined in the taxonomy are scarce. Examining non-specific antisocial behaviour, meta-analyses of structural neuroimaging data on children, adolescents, and adults with antisocial behaviour reported smaller grey matter volume, surface area, and cortical thickness (as well as atypical brain function) in the ventromedial, orbitofrontal, and dorsolateral prefrontal cortex, anterior and posterior cingulate cortex, and temporal cortex regions supporting executive function, motivation, and affect regulation.^12^, ^13^ A handful of neuroimaging comparisons between young people with childhood-onset and adolescence-onset conduct problems largely reported structural differences in the amygdala and insula,^13^ but these studies were cross-sectional, used small, unrepresentative middle-class samples, and have been defined by clinical classifications of antisocial behaviour based on retrospective recall.^14^, ^15^ No neuroimaging studies with antisocial behaviour groups defined by data-driven statistical models tracking prospective repeated measures of antisocial behaviour from childhood to adulthood exist.

We aimed to address the questions of whether previously reported structural brain differences linked to antisocial behaviour predominantly characterise individuals on the life-course-persistent trajectory, as hypothesised in the developmental taxonomy theory, and whether there are structural brain differences associated with life-course-persistent antisocial behaviour that have not been detected in previous small, cross-sectional studies. We focused on two structural features of neocortex—surface area and cortical thickness—making it possible to assess whether either of these measures was more indicative of antisocial behaviour pathology or differentially associated with antisocial behaviour trajectories. Our focus reflects both the preferential role of neocortical circuits in supporting higher-order executive processes implicated in neuropsychological deficits associated with life-course-persistent antisocial behaviour^15^, ^16^ and the preponderance of previous psychiatric neuroimaging findings in cortical regions.^17^ We hypothesised that individuals on the life-course-persistent but not adolescence-limited trajectories would show smaller surface area and cortical thickness than those with no history of persistent antisocial behaviour.

## Methods

### Study design and participants

Participants belonged to the Dunedin Study,^18^ a longitudinal investigation of health and behaviour in a population-representative birth cohort of 1037 individuals (91% of 1139 eligible births) born between April 1, 1972, and March 31, 1973, in Dunedin, New Zealand, who were eligible if they were resident in the province and participated in the first assessment at 3 years of age. The cohort is 93% white, and matches the New Zealand population on socioeconomic, health, and education measures.^18^ Assessments were done at birth and at 5, 7, 9, 11, 13, 15, 18, 21, 26, 31, 38, and 45 years of age, when 938 (94%) of the 997 participants still alive took part. Each participant attended the research unit at the University of Otago (Dunedin, New Zealand) for 1·5 days of data collection. Eligible participants completed MRI scanning and had been previously subtyped as exhibiting life-course-persistent, adolescence-limited, or no history of persistent antisocial behaviour (ie, a low trajectory group) based on informant-reported and self-reported conduct problems from the ages of 7 years to 26 years (appendix p 1).^19^

The relevant ethics committees approved each study phase, and informed consent was obtained from all participants.

### MRI data acquisition and processing

Participants were scanned using a Siemens Skyra 3T scanner (Siemens Healthcare, Erlangen, Germany) with a 64-channel head and neck coil. As part of the scanning protocol, high-resolution T1-weighted images, three-dimensional fluid-attenuated inversion recovery (FLAIR) images, and a gradient echo field map were obtained. Acquisition parameters and analysis preprocessing details are described in the appendix (p 3).

We analysed structural MRI data using the Human Connectome Project (HCP) minimal preprocessing pipeline, as detailed elsewhere.^20^ For each participant, mean cortical surface area and thickness were extracted from 360 parcels in the HCP-MPP1.0 parcellation.^21^ Outputs of the preprocessing pipeline were visually checked for accurate surface generation by examining each participant's myelin map, pial surface, and white matter boundaries. Study personnel who processed the MRI images were masked to antisocial group membership.

### Statistical analysis

Participants who met MRI data quality control measures were included in analyses. Student's *t* tests and resulting p values were used to compare groups on demographic and cognitive variables. Odds ratios and associated CIs were calculated to compare groups on psychiatric diagnoses at 45 years of age. First, we used linear estimated ordinary least squares regressions to compare each antisocial trajectory group (life-course persistent and adolescence limited) with the low trajectory group to examine whether antisocial behaviour was related to abnormalities in global surface area and mean cortical thickness. Next, we used parcel-wise linear regressions to identify antisocial trajectory group differences in surface area and cortical thickness in each of the 360 regions comprising the parcellation scheme described previously.^21^ We corrected for multiple comparisons across the 360 tests using a false discovery rate procedure. Sex was included as a covariate in all analyses. Total surface area and mean cortical thickness were not included as covariates in parcel-based analyses because we examined specific rather than relative regional associations as well as regional contributions to general cortex-wide effects, but we did exploratory analyses controlling for global values. Moreover, secondary analyses were done controlling for IQ, socioeconomic status, total intracranial volume, and head-injury history, and excluding individuals diagnosed with schizophrenia. All analyses were checked for reproducibility by an independent data analyst, who recreated the code by working from the manuscript and applied it to a fresh copy of the dataset. p values of less than 0·05 were considered to be significant.

All analyses were done with R, version 3.4.1.

### Role of the funding source

The funders had no input in study design, data collection, data analysis, data interpretation, writing of the report, or the decision to submit for publication. All authors had access to study data. The corresponding author had final responsibility for the decision to submit for publication.

## Results

**Figure 1:**
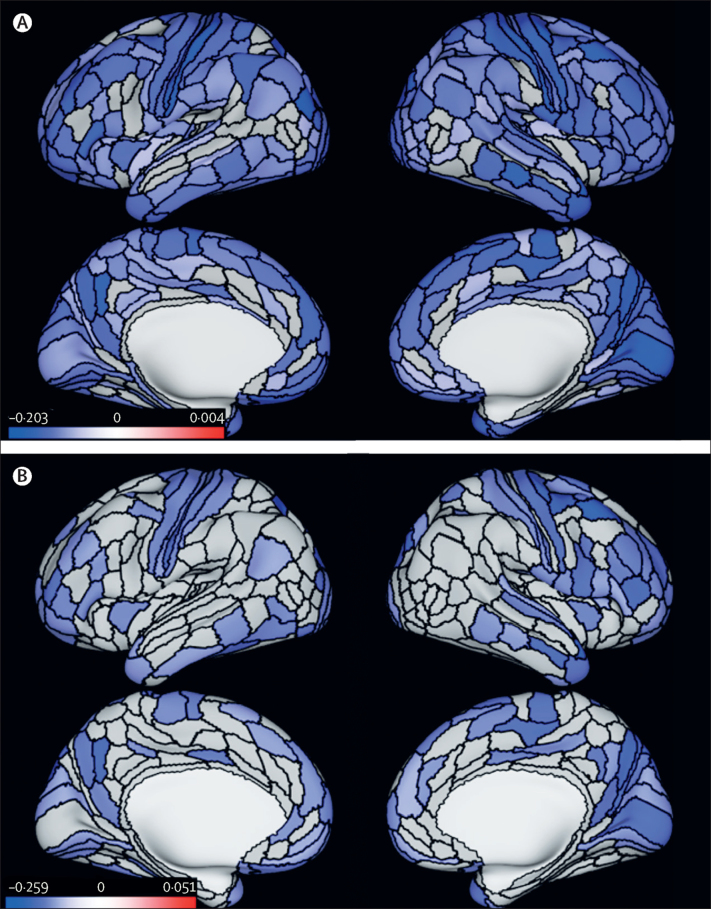
Differences in parcel-wise surface area between antisocial behaviour trajectory groups and the low antisocial behaviour group (A) Parcel-wise regions (in blue) with significantly smaller surface area in the life-course-persistent antisocial behaviour group than in the low group. (B) Parcel-wise regions (in blue) with significantly smaller surface area in the life-course-persistent antisocial behaviour group than in the adolescence-limited antisocial behaviour group. All results are controlled for sex and false discovery rate corrected, p<0·05. Colour bars represent z statistic values.

**Figure 2:**
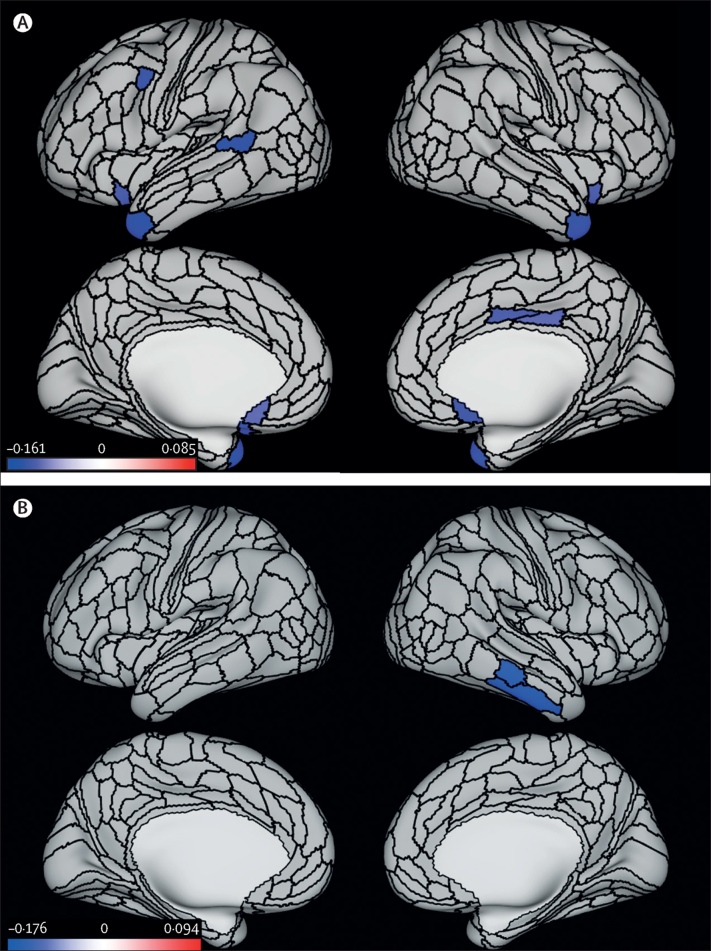
Differences in parcel-wise cortical thickness between the two antisocial behaviour trajectory groups and the low antisocial behaviour group (A) Parcel-wise regions (in blue) with significantly thinner cortex in the life-course-persistent antisocial behaviour group than in the low group. (B) Parcel-wise regions (in blue) with significantly thinner cortex in the adolescent-limited antisocial behaviour than in the low group. All results are controlled for sex and false discovery rate corrected, p<0·05. Colour bars represent z statistic values.

The most recent phase of data collection was at 45 years of age, completed in April, 2019. Of the participants with available structural MRI data, four were excluded because of major incidental findings or previous injuries (eg, tumours or extensive damage to the brain or skull), nine because of missing FLAIR or field map scans, one because of poor surface mapping, and ten because of other quality control issues. Of the 672 participants who had both usable MRI data and were subtyped in trajectory groups, 80 (12%) were classified as having life-course-persistent antisocial behaviour, 151 (23%) as having adolescence-limited antisocial behaviour, and 441 (66%) as having low antisocial behaviour. These proportions resemble those originally identified within the full cohort, including individuals who did not undergo MRI (124 [12%] of 1033 with life-course-persistent, 249 [24%] of 1033 with adolescence-limited, and 674 [65%] of 1033 with low antisocial behaviour).^19^ Complete cohort details and attrition analyses showing that participants in the age 45 years data collection group represent the original cohort are reported in the appendix (p 2). Demographic, cognitive, and psychiatric characteristics of the groups are described in table 1 and the appendix (pp 1–2). Participants in both antisocial behaviour groups grew up in lower socioeconomic backgrounds, performed more poorly on cognitive tests, and had higher levels of psychopathology than did those in the low antisocial behaviour group (table 1). The most common current psychiatric diagnoses at 45 years of age were major depressive disorder in the life-course-persistent group and anxiety in the adolescent-limited and low groups. The least common diagnoses were mania in the life-course-persistent and adolescence-limited groups and schizophrenia groups. Most cognitive and psychiatric measures were more significantly impaired in the life-course-persistent relative to the adolescence-limited group (table 1). Analyses of an additional childhood-limited group are reported in the appendix (p 4). Participants in the life-course-persistent, but not those in the adolescence-limited antisocial behaviour group, had smaller global surface area than did participants in the low group (standardised β=–0·18 [95% CI −0·24 to −0·11], p<0·0001, for the life-course-persistent *vs* low group; standardised β=–0·06 [95% CI −0·12 to 0·00], p=0·071, for the adolescent-limited *vs* low group; table 2). The life-course-persistent group also showed smaller mean global surface area than the adolescence-limited group (standardised β=–0·17 [95% CI −0·26 to −0·07], p=0·0008; table 2). Analyses showed widely distributed patterns of smaller surface area across 282 of 360 parcels in participants with life-course-persistent antisocial behaviour than in those in the low group (figure 1A). Similarly, comparisons of parcel-wise surface area between the life-course-persistent and adolescence-limited antisocial behaviour groups showed that the life-course-persistent group had smaller surface area in 125 parcels and largely in the same areas compared with the low group, although some of the parcel-wise differences from the comparison with the low group were not significant (figure 1B). No significant parcels were seen in the comparison between the adolescence-limited antisocial group and the low group (data not shown). Participants in the life-course-persistent and adolescent-limited trajectory groups had lower mean cortical thickness than did those in the low group (standardised β=–0·10 [95% CI −0·19 to −0·02], p=0·020, for the life-course-persistent *vs* low group; standardised β=–0·08 [95% CI −0·16 to 0·00], p=0·039, for the adolescent-limited *vs* low group; table 2). Participants in the two antisocial groups did not differ significantly from each other. Parcel-wise analyses revealed that in comparison with the low group, participants in the life-course-persistent antisocial behaviour group had reduced cortical thickness in 11 parcels, including left lateral prefrontal cortex and superior temporal gyrus, right posterior cingulate cortex, and bilateral ventromedial prefrontal and orbitofrontal cortices, and temporal pole, which are regions associated with executive function, affect regulation, and motivation (figure 2A). By contrast, parcel-wise analyses comparing the low group with the adolescence-limited group revealed only two parcels with lower cortical thickness in the right inferior and middle temporal gyrus (figure 2B). Comparisons of parcel-wise cortical thickness between the life-course-persistent and adolescence-limited groups did not yield significant differences.

**Table 1:** Demographic, cognitive, and psychiatric characteristics

		Antisocial trajectory groups*			**Comparisons between trajectory groups**		
		Life-course persistent (n=80)	Adolescence limited (n=151)	Low (n=441)	Life-course persistent *vs* low	Adolescence limited *vs* low	Life-course persistent *vs* adolescence limited
**Demographic factors**							
Sex					1·58 (0·98–2·56)	1·32 (0·91–1·91)	1·20 (0·69–2·07)
	Male	47 (59%)	82 (54%)	209 (47%)	..	..	..
	Female	33 (41%)	69 (46%)	232 (53%)	..	..	..
Socioeconomic status in childhood†		3·03 (0·98)	3·58 (1·08)	4·03 (1·12)	p<0·0001	p<0·0001	p=0·0002
Socioeconomic status at 45 years of age		2·64 (1·47)	3·60 (1·66)	4·03 (1·36)	p<0·0001	p=0·0013	p<0·0001
Brain health at 3 years of age‡		−0·403 (1·04)	0·156 (0·80)	0·215 (0·86)	p<0·0001	p=0·459	p<0·0001
WISC-R intelligence quotient in childhood		93·24 (13·70)	101·21 (14·00)	103·81 (13·10)	p<0·0001	p=0·0395	p<0·0001
WAIS-IV intelligence quotient at 45 years of age		90·09 (12·83)	98·59 (14·52)	103·64 (14·38)	p<0·0001	p=0·0002	p<0·0001
Lifetime history of head injury§		9 (11%)	28 (19%)	44 (10%)	1·14 (0·54–2·45)	2·05 (1·23–3·44)	0·56 (0·25–1·25)
**Lifetime psychopathology factor scores**¶							
p factor, general psychopathology		112·58 (16·81)	105·23 (14·13)	95·38 (12·96)	p<0·0001	p<0·0001	p=0·0005
Internalising factor		110·46 (16·98)	103·21 (14·02)	96·70 (13·54)	p<0·0001	p<0·0001	p=0·0006
Externalising factor		117·13 (15·94)	109·92 (13·25)	93·73 (11·91)	p<0·0001	p<0·0001	p=0·0003
Thought disorder factor		113·15 (16·43)	105·69 (13·93)	95·26 (12·99)	p<0·0001	p<0·0001	p=0·0003
**Psychiatric diagnosis at 45 years of age**							
Schizophrenia (lifetime)		9 (11%)	10 (7%)	2 (<1%)	27·82 (5·89–131·43)	15·57 (3·37–71·89)	1·79 (0·70–4·60)
Mania (lifetime)		2 (3%)	2 (1%)	7 (2%)	1·59 (0·32–7·80)	0·83 (0·17–4·05)	1·91 (0·26–13·82)
Major depressive episode		25 (31%)	25 (17%)	54 (12%)	3·25 (1·87–5·64)	1·42 (0·85–2·37)	2·29 (1·21–4·34)
Anxiety disorder		22 (28%)	31 (21%)	74 (17%)	1·91 (1·10–3·31)	1·28 (0·80–2·04)	1·49 (0·80–2·81)
Alcohol dependence		16 (20%)	25 (17%)	37 (8%)	2·72 (1·43–5·18)	2·16 (1·25–3·73)	1·26 (0·63–2·53)
Cannabis dependence		9 (11%)	6 (4%)	2 (<1%)	27·76 (5·88–131·13)	9·06 (1·81–45·39)	3·06 (1·05–8·94)
Drug (other) dependence		10 (13%)	10 (7%)	5 (1%)	12·43 (4·13–37·44)	6·17 (2·07–18·36)	2·01 (0·80–5·06)

Data are odds ratio (95% CI), n (%), or mean (SD) unless otherwise stated. p values were calculated with the Student's *t* test. WAIS-IV=Wechsler Adult Intelligence Scale-IV. WISC-R=Wechsler Intelligence Scale for Children—Revised.

* Growth-mixture modelling was applied to derive developmental subtypes of antisocial behaviour, within sex, as previously published.^19^

† Socioeconomic status in childhood was assessed as the highest of father's or mother's occupation.

‡ Brain health, a global index of the child's early neurocognitive status,^22^, ^23^ was assessed when each child participated in a 45 min examination including assessments of neurological soft signs, intelligence, receptive language, and motor skills, after which the examiners (having no previous knowledge of the child) rated each child's behaviour. Using this information, a summary factor score was created via confirmatory factor analysis (standardised to mean 100 [SD 15]).

§ Self-reported head injury requiring hospital treatment assessed at 45 years of age during a clinical interview.

¶ Dimensional factor scores for internalising and externalising symptoms and general liability for psychopathology were derived using confirmatory factor analysis as reported in Romer and colleagues.^24^ Briefly, DSM-defined psychopathology symptoms were repeatedly assessed through private structured interviews using the Diagnostic Interview Schedule at the ages of 18, 21, 26, 32, 38, and 45 years, and factor scores were derived from a validated bi-factor model.^25^ Psychopathology factor scores have a mean 100 (SD 15).

**Table 2:** Mean global surface area and mean cortical thickness, and antisocial group comparisons

	**Global surface area**	**Cortical thickness**
Life-course-persistent antisocial behaviour group	Mean 181 204·72 mm^2^ (SD 17 382·35)	Mean 2·54 mm (SD 0·10)
Adolescence-limited antisocial behaviour group	Mean 186 208·29 mm^2^ (SD 16 809·61)	Mean 2·55 mm (SD 0·09)
Low antisocial behaviour group	Mean 187 000·77 mm^2^ (SD 16 135·46)	Mean 2·56 mm (SD 0·08)
Life-course-persistent antisocial behaviour group *vs* low antisocial behaviour group	Standardised β=−0·18 (95% CI −0·24 to −0·11); p<0·0001	Standardised β=−0·10 (95% CI −0·19 to −0·02); p=0·020
Adolescence-limited antisocial behaviour group *vs* low antisocial behaviour group	Standardised β=–0·06 (95% CI −0·12 to 0·00); p=0·071	Standardised β=–0·08 (95% CI −0·16 to 0·00); p=0·039
Life-course-persistent antisocial behaviour group *vs* adolescence-limited antisocial behaviour group	Standardised β=–0·17 (95% CI −0·26 to −0·07); p=0·0008	Standardised β=–0·04 (95% CI −0·17 to 0·09); p=0·56

In the exploratory analyses, p values derived through permutation testing for these comparisons for both surface area and cortical thickness are similar to those reported in table 2 (appendix p 11). There were no significant differences in parcel-wise surface area for participants with adolescence-limited antisocial behaviour compared with the low group. Forest plots showing parcel-wise effect sizes of surface area and cortical thickness between all three groups for all 360 parcels are presented in the appendix (p 5). Exploratory parcel-wise analyses with family-wise-error correction show that although some parcels that were significant using a false discovery rate procedure did not survive family-wise error correction, results led to similar overall inference with regard to the observed pattern of findings. The differences in surface area and cortical thickness were greater between individuals the life-course-persistent group versus the low group than between the adolescence-limited group versus the low group (appendix p 12).

Secondary analyses including IQ, socioeconomic status, total intracranial volume, and head-injury history, and excluding individuals diagnosed with schizophrenia left findings largely unaltered (appendix p 13).

## Discussion

Our analyses revealed findings in line with the developmental taxonomy theory. First, we found that smaller surface area and thinner cortex in brain regions associated with executive function, motivation, and affect regulation previously implicated in studies of antisocial behaviour were, as hypothesised, specific to the life-course-persistent group. Second, the observed pattern of smaller surface area specific to the life-course-persistent group was much broader than previously reported in cross-sectional studies of antisocial behaviour. Third, both antisocial behaviour trajectory groups had features of reduced cortical thickness compared with the low group without antisocial behaviour, but in different areas of the brain. In the adolescence-limited group, the pattern was limited to two regions in the right temporal lobe that have not been consistently implicated in previous studies of antisocial behaviour, whereas in the life-course-persistent group, the pattern extended to paralimbic frontal and temporal regions that have been previously implicated in antisocial behaviour.^16^ When compared directly, the two antisocial behaviour groups were distinguished on the basis of patterns of broadly smaller surface area in the life-course-persistent group relative to the adolescence-limited group.

So far, the majority of research comparing life-course-persistent and adolescence-limited antisocial behaviour (or proposed proxies for these subtypes) has not used optimal study designs for investigating the underlying brain correlates of antisocial behaviour subtypes as originally delineated by the developmental taxonomy theory.^2^, ^14^ Our research is a step towards improving study design for investigating developmental trajectories of antisocial behaviour by using a population-representative cohort with repeated measures of antisocial behaviour beginning in childhood to define trajectories of antisocial behaviour. Because we assessed brain surface morphometry differences between these groups only at the age of 45 years, we were unable to determine whether these brain features relate to genetic or other early life risk factors that might lead to an antisocial lifestyle, or whether they are a consequence of a persistent antisocial lifestyle. Moreover, the investigation of subcortical brain regions was outside the scope of our study, but this is important for future work given the implication of subcortical structural abnormalities when childhood-onset and adolescent-onset antisocial groups have been compared previously.^13^

Early-onset antisocial behaviour, which characterises the life-course-persistent group, is strongly heritable, and the people with life-course-persistent antisocial behaviour are also more likely to have experienced childhood adversity.^26^ Individual differences in surface area and cortical thickness are also highly heritable, and early childhood adversity is associated with smaller surface area and thinner cortex,^27^ which is the pattern we observed in individuals with life-course-persistent antisocial behaviour. It is possible that smaller surface area and thinner cortex in the life-course-persistent group, affected by genetic or environmental risk, might have predated (and indeed been a risk factor for) an antisocial lifestyle. Participants with life-course-persistent antisocial behaviour had poor neuropsychological function in childhood that predicted their later antisocial behaviour,^5^, ^7^ although this finding does not constitute causal evidence that the structural brain differences reported here predated later antisocial behaviour. Moreover, although the Dunedin Study is a population-representative cohort, 93% of the sample is white. Further work is needed to evaluate generalisability of the results to other populations.

It is also possible that the observed structural brain features arose from a lifetime of confounding risk factors (eg, substance abuse, low IQ, and psychiatric comorbidity) and are therefore a consequence of the life-course-persistent antisocial lifestyle. Secondary analyses including IQ, socioeconomic status, total intracranial volume, and head-injury history, and excluding individuals diagnosed with schizophrenia left findings largely unaltered. It was not meaningful to separate these risk factors because they have been shown to be virtually inherent core characteristics of the life-course-persistent trajectory (table 1).^6^ Excluding individuals with features such as low IQ (or controlling for them statistically) would negate the core life-course-persistent phenotype. In line with this core phenotype theory, common genetic and environmental factors also predispose to both antisocial behaviour and substance use disorders.^9^ Prospective collection of neuroimaging data beginning in childhood, alongside collection of behavioural, genetic, and environmental measures, is crucial for understanding causal temporal order. Ideally, such data will be collected in samples that are sufficiently large to test for sex differences, which we were unable to do in the current study.

Several explanations could account for the pervasive and non-specific signature of smaller surface area associated with life-course-persistent antisocial behaviour. Surface area and cortical thickness are under distinct genetic control,^28^ indicating that separate underlying processes give rise to individual differences in these metrics. Moreover, surface area is more strongly heritable and prone to disruption by early adversity, a known risk factor for life-course-persistent antisocial behaviour.^29^ This evidence reinforces the importance of incorporating both of these measures when considering links between phenotypes of brain structure and behaviour.

In contrast to the broad patterns of smaller surface area, lower cortical thickness associated with life-course-persistent antisocial behaviour was more circumscribed. Differences in the structure of the ventromedial prefrontal cortex and posterior cingulate have been previously shown in adults with persistent antisocial behaviour^30^ and in youths with conduct problems.^13^ The ventromedial prefrontal and orbitofrontal cortices are key for processes that have been shown to be impaired in early-onset conduct disorder, including integration of affective input from subcortical structures, as well as monitoring top-down executive control.^31^ Lower cortical thickness of the posterior cingulate cortex has been observed in conduct disorder,^32^ and abnormal activation of this region has been observed in conduct-disordered youths during tasks of inhibitory control and risk taking,^33^ which are thought to be most impaired in life-course-persistent antisocial behaviour.^9^ Moreover, smaller ventromedial prefrontal and orbitofrontal cortices have been linked to aggression and impulsivity in animal studies and in lesion and neuroimaging studies in humans, as well as in adults with antisocial and violent behaviour, and psychopathy,^12^, ^30^ in line with key behavioural and personality characteristics of life-course-persistent antisocial behaviour. Taken together, the pattern of regional cortical thinning agrees with existing evidence that life-course-persistent antisocial behaviour is linked to structural abnormalities in regions functionally associated with prosocial behaviour, although the direct mapping of structural brain differences onto cognition and behaviour should be interpreted with caution.

Participants with life-course-persistent antisocial behaviour also had thinner cortex in the superior temporal gyrus and temporal pole than those without antisocial behaviour. Reduced thickness in the superior temporal gyrus of youths with conduct disorder has been reported previously and is hypothesised to be related to social-cognitive impairments that are pronounced in people with early-onset conduct disorder.^16^ These findings were not observed in individuals with adolescence-limited antisocial behaviour, who showed thinner cortex only in the middle and inferior temporal gyrus, relative to those without antisocial behaviour. These respective findings in life-course-persistent and adolescence-limited antisocial behaviour are in contrast to previous studies postulating that cortical thinning in paralimbic regions reflects a non-specific effect of antisocial behaviour^34^ and provide novel evidence for differentiating these subtypes. However, in comparisons of cortical thickness, the adolescence-limited group did not significantly differ from the life-course-persistent group, possibly because tests comparing the two antisocial behaviour groups included fewer individuals and had less statistical power to detect group differences. Alternatively, it is possible that adolescence-limited individuals are not entirely risk free in terms of structural brain features associated with antisocial behaviour, but prospective neuroimaging is needed to confirm this. Regardless, although previous studies cannot be mapped directly onto our findings with the life-course-persistent group, we can speculate that atypical brain development that compromises social-cognitive processing probably increases the risk of persistent antisocial behaviour.^8^

The developmental taxonomy theory of antisocial behaviour, first posed 25 years ago, has influenced juvenile justice policy in the UK, USA, and elsewhere^8^ to recognise that individual development is one driver of serious recidivistic crime, and to appreciate heterogeneity within antisocial individuals and offenders. Our study provides initial evidence of clear structural brain differences between individuals with life-course-persistent antisocial behaviour (who are fewer in number, with poorer prognosis and more urgent treatment need) and those on the adolescence-limited trajectory (who are more numerous with a relatively good prognosis). Findings highlight the importance of using prospective longitudinal data when studying patterns of antisocial behaviour development, with a clear need for longitudinal studies with multiple measurements of behaviour, brain, genetics, and environment to understand how life-course-persistent antisocial behaviour unfolds.

## Data sharing

The datasets reported in the current article are not publicly available because of lack of informed consent and ethical approval, but are available on request by qualified scientists. Requests require a concept paper describing the purpose of data access, ethical approval at the applicant's university, and provision for secure data access (https://moffittcaspi.trinity.duke.edu/research). We offer secure access on Duke University (Durham, NC, USA), University of Otago (Dunedin, New Zealand), and King's College London (London, UK) campuses. All data analysis scripts and results files are available for review.
