# Supplementary material for: Associations between life-course-persistent antisocial behaviour and brain structure in a population-representative longitudinal birth cohort
Source: Lancet Psychiatry. 2020 Mar;7(3):245–53. doi: 10.1016/S2215-0366(20)30002-X (PMC7033555; doi:10.1016/S2215-0366(20)30002-X)
Supplement: Supplementary appendix [file mmc1.pdf]

THE LANCET
Psychiatry

Supplementary appendix

This appendix formed part of the original submission and has been peer reviewed. We post it as supplied by the authors.

Supplement to: Carlisi CO, Moffitt TE, Knodt AR, et al. Associations between life-course-persistent antisocial behaviour and brain structure in a population-representative longitudinal birth cohort. *Lancet Psychiatry* 2020; published online Feb 17. http://dx.doi.org/10.1016/S2215-0366(20)30002-X.

Associations between life-course-persistent antisocial behavior and brain structure in a longitudinal birth cohort – Supplementary Materials

METHODS

Dunedin Study members

The cohort represents the full range of socioeconomic status on NZ's South Island and, as adults, matches the NZ National Health and Nutrition Survey on adult health indicators (e.g., BMI, smoking, GP visits) and the NZ census of citizens of the same age on educational attainment. The cohort is primarily white (964 of 1,037 Study members; 93%), matching South Island demographics.

Adult measures of demographic, cognitive and psychiatric symptoms

Socioeconomic status was assessed using a six-point scale developed by Elley & Irving.[1] This measure places each occupation in to one of six categories based upon educational levels and income associated with that occupation in data from the New Zealand census. The scale ranges from 1 = "unskilled laborer" to 6 = "professional."

Cognitive functioning at age 45 was assessed with the Wechsler Adult Intelligence Scale-IV,[2] which measures the Intelligence Quotient (IQ) and four specific domains of cognitive function: Verbal Comprehension, Perceptual Reasoning, Working Memory, and Processing Speed. Full-scale IQ was standardized to mean 100 ± 15. Study members were also tested with an additional suite of measures of vocabulary, memory, and executive functioning.

History of head injury (self-reported head injury requiring hospital treatment), as well as *current diagnosis* of schizophrenia, mania, anxiety disorder, major depressive episode, alcohol or drug dependence were assessed at age 45 during clinical interview.

Dimensional factor scores for internalizing and externalizing symptoms as well as and general liability for psychopathology were derived using confirmatory factor analysis as reported in Romer et al.[3] Briefly, DSM-defined psychopathology symptoms were repeatedly assessed through private structured interviews using the Diagnostic Interview Schedule at ages 18, 21, 26, 32, 38, and 45 years, and factor scores were derived from a validated bi-factor model.[4]

Childhood measures of demographic information and cognitive functioning

Socioeconomic status in childhood was assessed as the highest of father's or mother's occupation using a 6-point scale for New Zealand.[1]

'*Brain health'*, a global index of the child's early neurocognitive status[5,6] was assessed at age 3, when each child participated in a 45-minute examination including assessments of neurological soft signs, intelligence, receptive language, and motor skills, after which the examiners (having no prior knowledge of the child) rated each child's behavior. Using this information, a summary factor score was created via confirmatory factor analysis.

Childhood cognitive function was assessed via IQ scores which were derived in late childhood (ages 7, 9, and 11 years), when study members were administered the Wechsler Intelligence Scale for Children–Revised (WISC-R).[7] Scores from the three WISC-R administrations were averaged to yield a single, reliable measure of cognitive function.

Assessment of antisocial behaviour

Antisocial behavior was assessed prospectively at ages 7, 9, 11, 13, 15, 18, 21, and 26 via mother and teacher reports of conduct problems in childhood and adolescence, self-reports in adolescence, and informant and self-reports in young adulthood.[8–10] Informants were identified by Study members as "three people who know me well" and were sent postal questionnaires. At least one of these questionnaires were returned for 97% of Study members. Six key facets of antisocial behavior were scored as present or absent at each age: physical fighting, bullying, destroying property, lying, truancy (or chronic work absenteeism), and stealing. Growth mixture modelling was applied to these data, and developmental subtypes of antisocial behavior were identified within sex, as previously published.[10] These groups were subsequently followed; life-course-persistent individuals

were convicted five times on average between age 26 and 38, compared to once for the adolescence-limited group and 0.31 times for the low antisocial group.[9]

A group previously identified as brief childhood-limited conduct problems[10] was not studied here, as this group was not posited in the 1993 Taxonomy[11] and has not been a focus of recent investigations of antisocial behavior. Including this group would not follow a hypothesis-driven approach, and interpretation of results would not be guided by *a priori* predictions regarding brain structure abnormalities in this group.

Age-45 attrition analysis

We conducted an attrition analysis using childhood IQ, childhood SES and p-factor (using ages 18-45 year data) to determine whether participants in the age-45 data collection were representative of the original cohort.

No significant differences in childhood IQ were found between the full cohort, those still alive, those seen at age-45 or those scanned at age-45. Those who were deceased by the age-45 data collection had significantly lower childhood IQ's than those who were still alive (t=2.09, p=0.04).

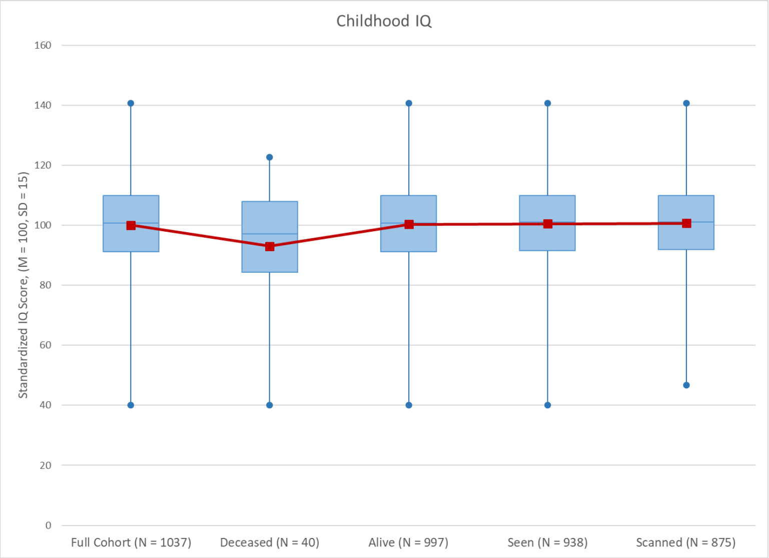

Figure S1. Attrition analysis of childhood IQ comparing age-45 study members with original cohort

No significant differences were found between the full cohort, those deceased, those alive, those seen at age-45 or those scanned at age-45 on childhood SES.

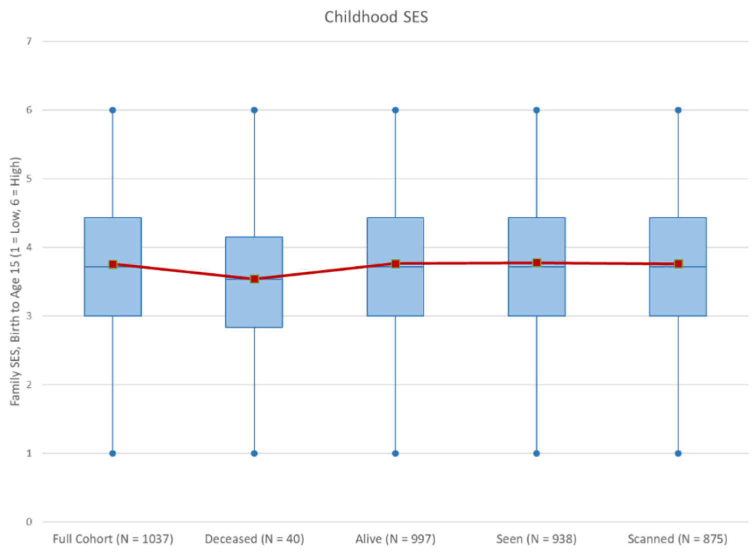

Figure S2. Attrition analysis of childhood SES comparing age-45 study members with original cohort

No significant differences in p-factor were found between the full cohort, those still alive, those seen at age-45 or those scanned at age-45. Those who were deceased by the age-45 data collection had significantly higher p-factor scores than those who were still alive (t=-2.86, p=0.004).

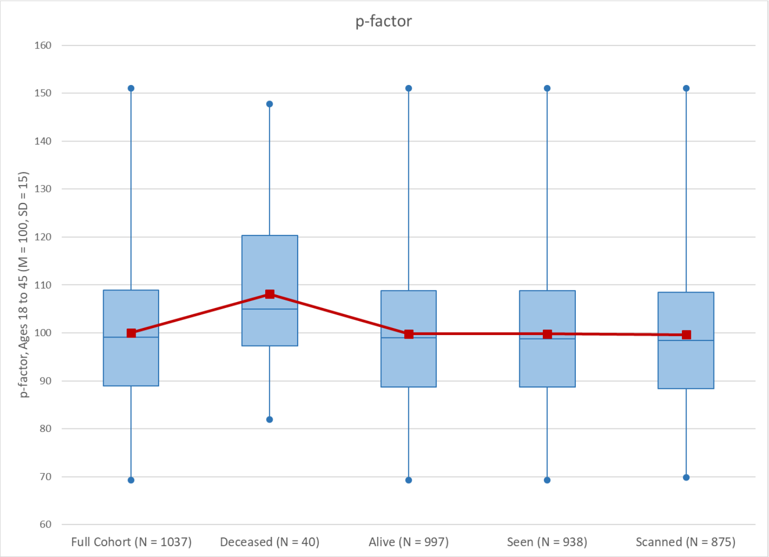

Figure S3. Attrition analysis of p-factor scores comparing age-45 study members with original cohort

MRI data acquisition

High resolution T1-weighted images were obtained using an MP-RAGE sequence with the following parameters: TR=2400ms; TE=1.98ms; 208 sagittal slices; flip angle, 9°; FOV, 224 mm; matrix=256×256; slice thickness=0.9mm with no gap (voxel size 0.9×0.875×0.875mm); total scan time=6min 52s. 3D fluid-attenuated inversion recovery (FLAIR) images were obtained with the following parameters: TR=8000ms; TE=399ms; 160 sagittal slices; FOV=240 mm; matrix=232×256; slice thickness=1.2mm (voxel size 0.9×0.9×1.2mm); and total scan time=5min 38s. Additionally, a gradient echo field map was acquired with the following parameters: TR=712ms; TE=4.92 and 7.38ms; 72 axial slices; FOV=200 mm; matrix=100×100; slice thickness=2.0mm (voxel size 2mm isotropic); total scan time = 2min 25s.

MRI data preprocessing

T1-weighted and FLAIR images were processed through the PreFreeSurfer, FreeSurfer, and PostFreeSurfer pipelines. Images were corrected for readout distortion using the gradient echo field map, coregistered, brain-extracted, and aligned together in native T1 space using boundary-based registration.[12] Images were then processed with a custom FreeSurfer recon-all pipeline optimized for structural MRI with higher resolution than 1mm isotropic. Finally, recon-all output were converted into CIFTI format and registered to common 32k_FS_LR mesh using MSM-sulc.[13]

RESULTS

Imaging results – comparisons with the childhood-limited group

Average values for cortical thickness and global surface area in the childhood-limited antisocial behavior group, along with comparisons of these metrics to the other antisocial groups and the low group, are presented in **Table S1**. The childhood-limited group differed only from the low group on global surface area.

Table S1. Mean average cortical thickness and global surface area for childhood-limited participants (N=186) and group comparisons

	**Global surface area**	**Average cortical thickness**
**Mean (SD)**	183330.92 (15568.80)	2.55 (.09)
**Childhood-limited vs. low**	Standardized β=-0.13<br>95% CI: -0.20 – -0.07<br>*p*<0.001	Standardized β=-0.06<br>95% CI: -0.14 – 0.02<br>*p*=0.13
**Childhood-limited vs. life-course-persistent**	Standardized β=-0.09<br>95% CI: -0.19 – 0.00<br>*p*=0.06	Standardized β=-0.06<br>95% CI: -0.18 – 0.06<br>*p*=0.29
**Childhood-limited vs. adolescent-limited**	Standardized β=0.08<br>95% CI: -0.01 – 0.16<br>*p*=0.07	Standardized β=-0.03<br>95% CI: -0.14 – 0.08<br>*p*=0.59

Parcel-wise analysis of surface area revealed that when compared to the low group, the childhood-limited group showed lower surface area in 248 parcels (**Figure S4**). No other group comparisons of surface area with the childhood-limited group revealed significant differences. Parcel-wise analysis of cortical thickness did not reveal any significant differences between the childhood-limited group and all other groups.

Figure S4. Differences in parcel-wise surface area between the childhood-limited antisocial group and the low-antisocial group

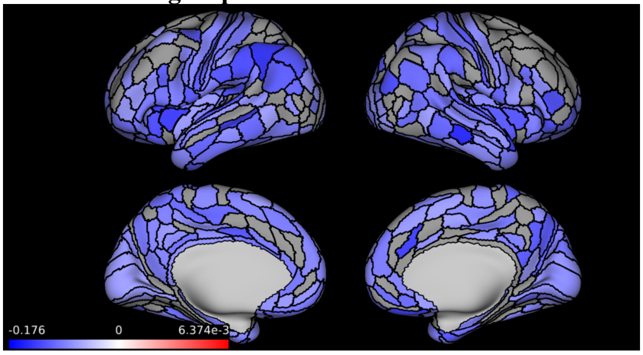

Figure S4. Differences in parcel-wise surface area between the childhood-limited antisocial behavior trajectory group and the low antisocial behavior group. Parcel-wise regions (in blue) represent areas that were significantly lower in cortical thickness in Study members with childhood-limited antisocial behavior. Not shown are the comparisons of the childhood-limited group to the life-course-persistent and adolescence-limited antisocial groups, as no significant parcels were observed. All results are controlled for sex and false discovery rate (FDR) corrected, *p*<0.05.

Forest plots of effect sizes for parcel-wise comparisons among the life-course-persistent and adolescence-limited antisocial groups and the low-antisocial group.

Forest plots show that even in parcels that did not reach statistical significance, effects generally echoed those observed in the significant findings, with surface area and cortical thickness comparatively lower in the life-course-persistent group relative to the low and adolescence-limited groups.

Figure S5. Forest plots of effect sizes comparing parcel-wise surface area between the life-course-persistent group and the low group

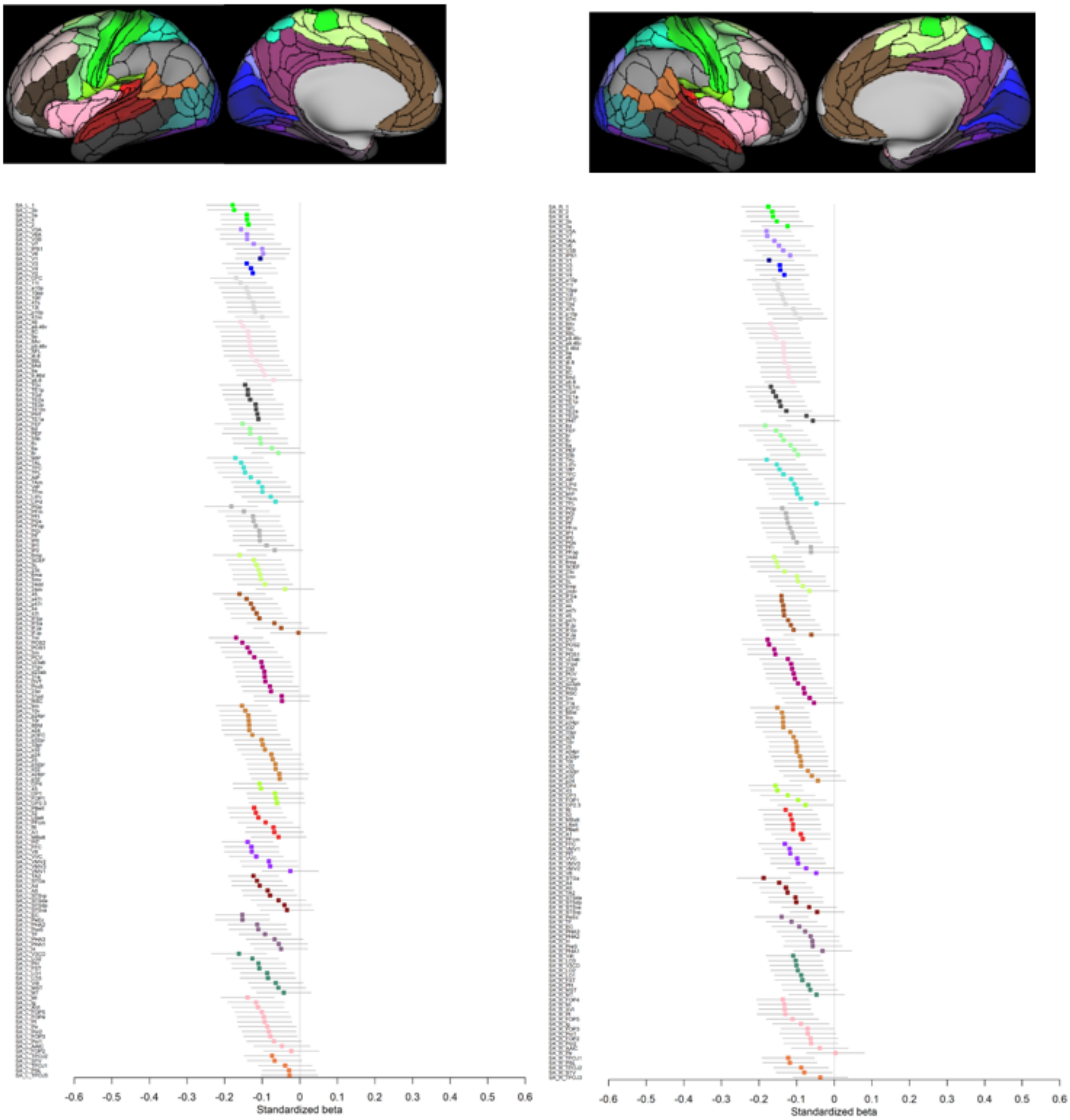

Figure S5. Forest plots of effect sizes comparing parcel-wise surface area between the life-course-persistent group and the low group. Forest plots showing parcel-wise effect sizes (represented by standardized beta values) for each parcel in the parcellation scheme denoted by the brain maps in this figure and outlined in Glasser et al., 2016[14]. Each parcel is colour-coded according to 22 broader regions based on anatomical and functional criteria outlined in Glasser et al., 2016[14]. Parcels are then sorted by magnitude of effect size within each region. The left side of the figure corresponds with the left hemisphere.

Figure S6. Forest plots of effect sizes comparing parcel-wise surface area between the adolescence-limited group and the low group

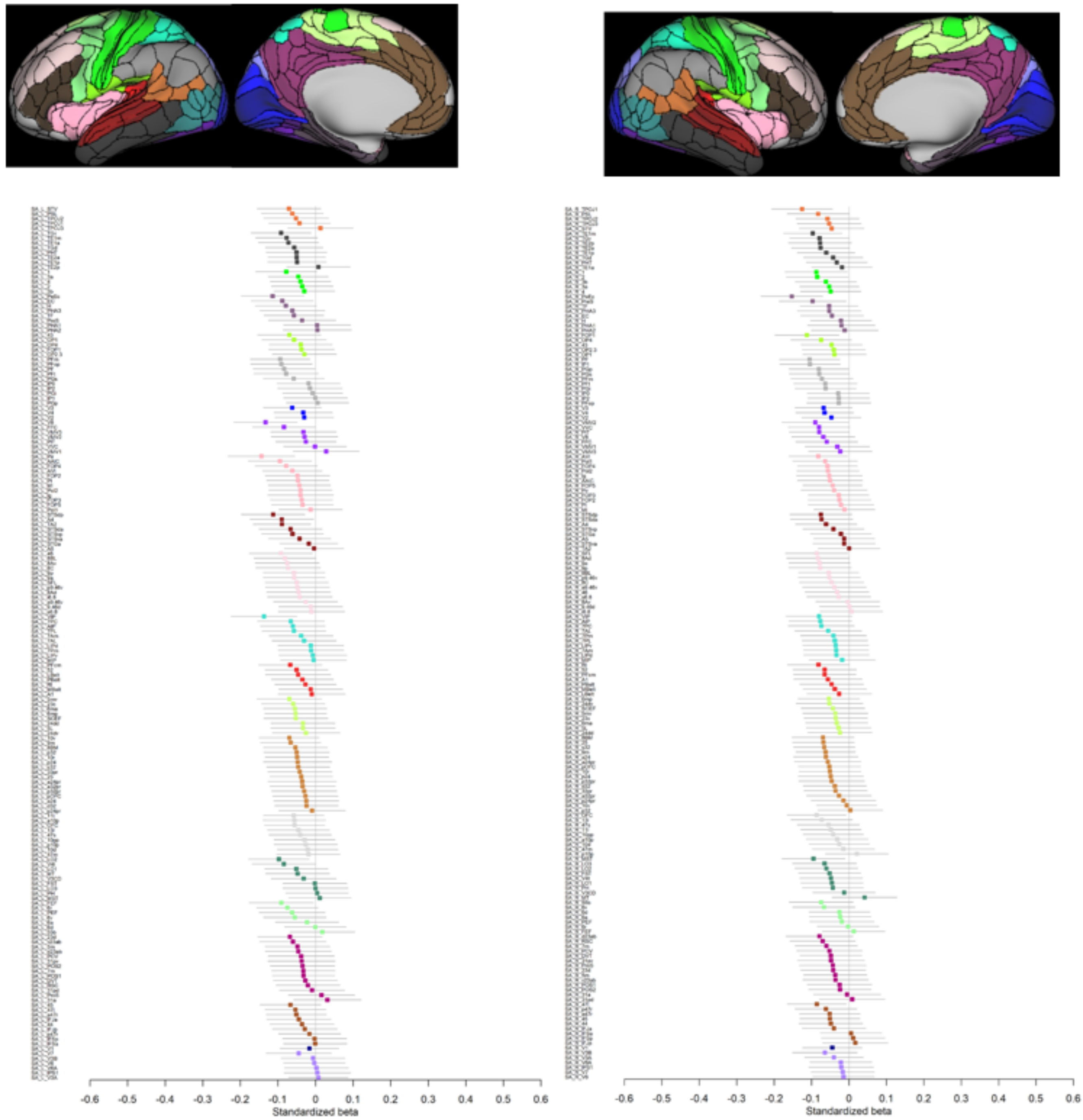

Figure S6. Forest plots of effect sizes comparing parcel-wise surface area between the adolescence-limited group and the low group. Forest plots showing parcel-wise effect sizes (represented by standardized beta values) for each parcel in the parcellation scheme denoted by the brain maps in this figure and outlined in Glasser et al., 2016[14]. Each parcel is colour-coded according to 22 broader regions based on anatomical and functional criteria outlined in Glasser et al., 2016[14]. Parcels are then sorted by magnitude of effect size within each region. The left side of the figure corresponds with the left hemisphere.

Figure S7. Forest plots of effect sizes comparing parcel-wise surface area between the life-course-persistent and adolescence-limited groups

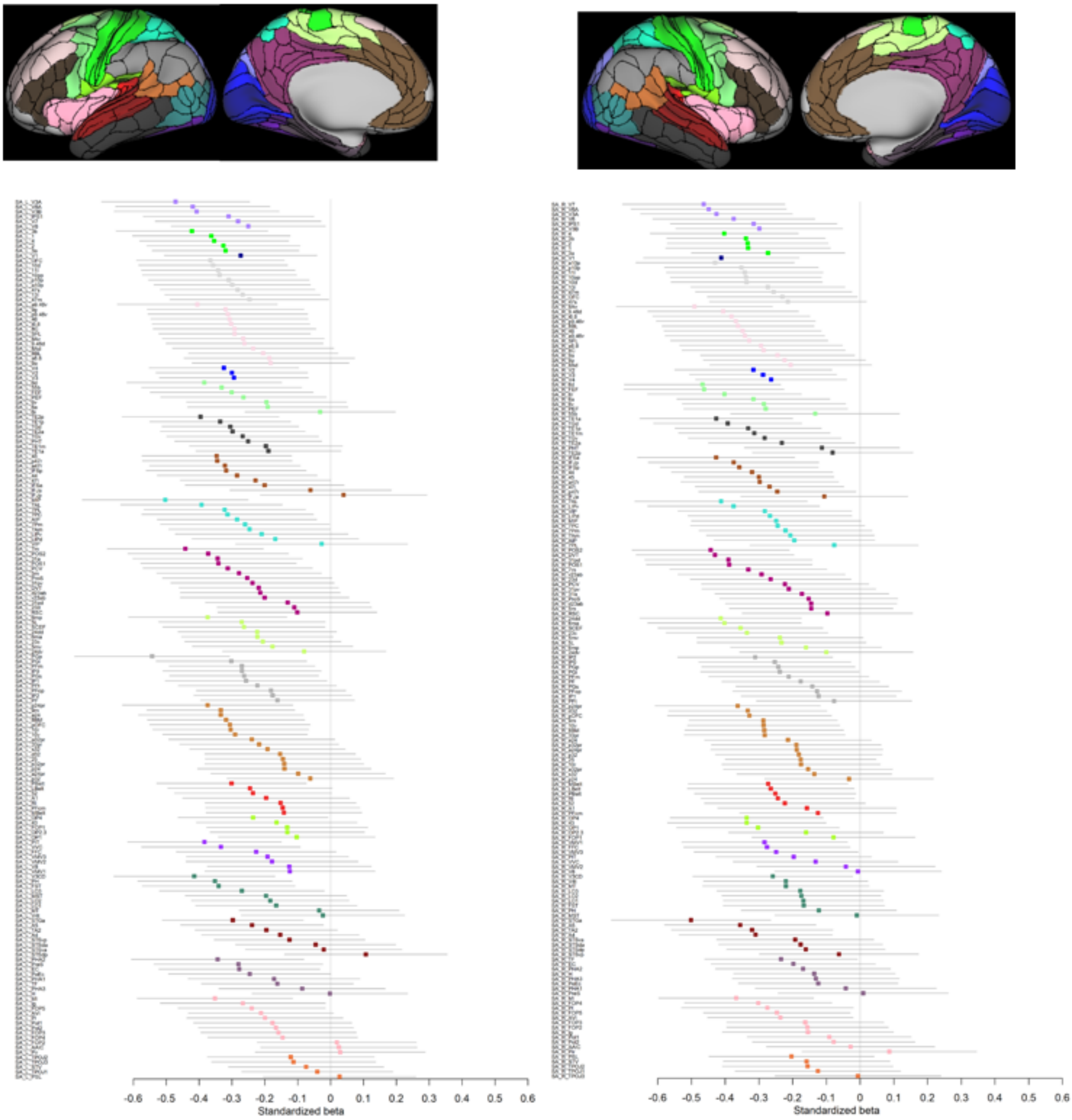

Figure S7. Forest plots of effect sizes comparing parcel-wise surface area between the life-course-persistent and adolescence-limited groups. Forest plots showing parcel-wise effect sizes (represented by standardized beta values) for each parcel in the parcellation scheme denoted by the brain maps in this figure and outlined in Glasser et al., 2016[14]. Each parcel is colour-coded according to 22 broader regions based on anatomical and functional criteria outlined in Glasser et al., 2016[14]. Parcels are then sorted by magnitude of effect size within each region. The left side of the figure corresponds with the left hemisphere.

Figure S8. Forest plots of effect sizes comparing parcel-wise cortical thickness between the life-course-persistent group and the low group

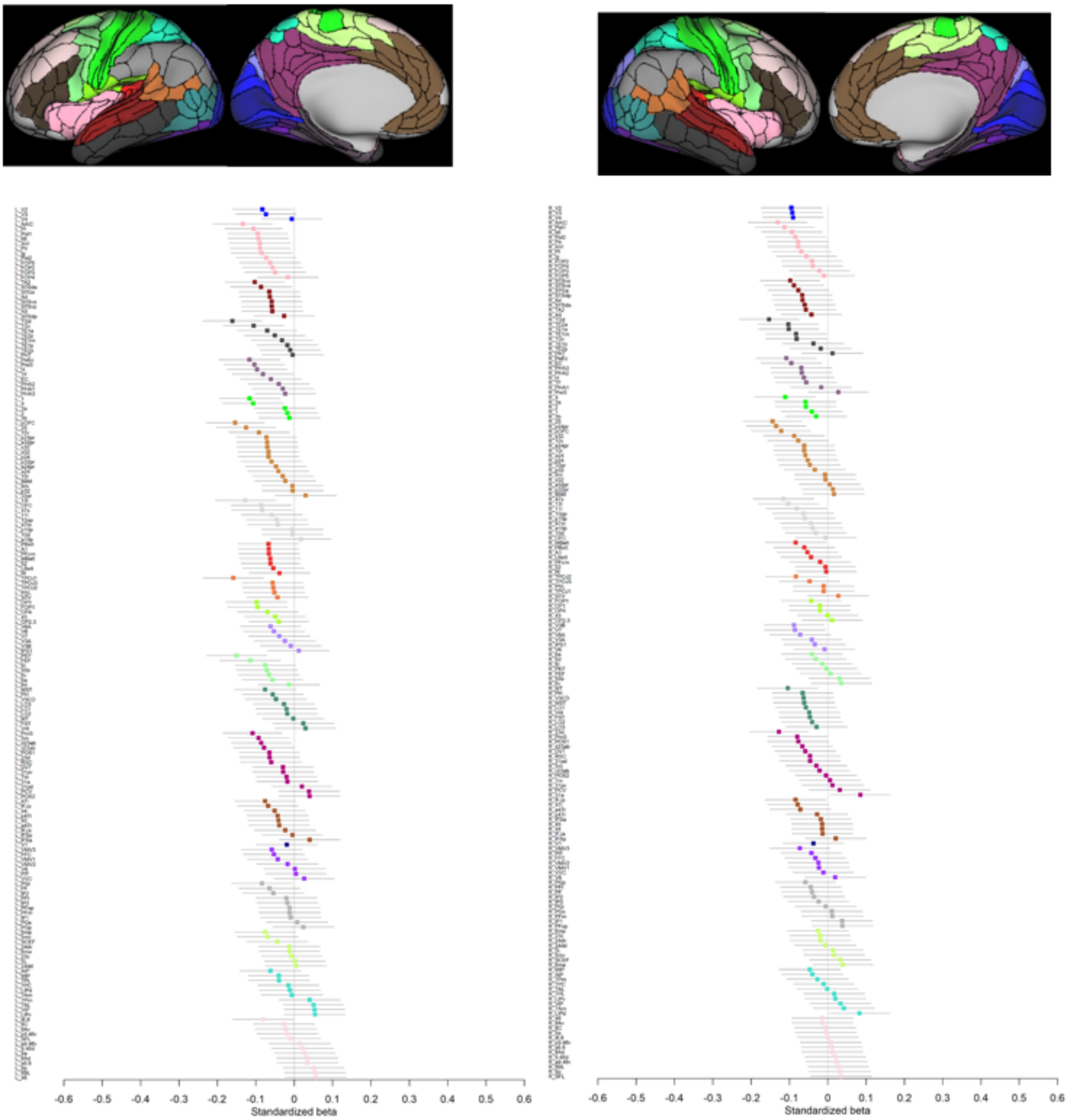

Figure S8. Forest plots of effect sizes comparing parcel-wise cortical thickness between the life-course-persistent group and the low group. Forest plots showing parcel-wise effect sizes (represented by standardized beta values) for each parcel in the parcellation scheme denoted by the brain maps in this figure and outlined in Glasser et al., 2016[14]. Each parcel is colour-coded according to 22 broader regions based on anatomical and functional criteria outlined in Glasser et al., 2016[14]. Parcels are then sorted by magnitude of effect size within each region. The left side of the figure corresponds with the left hemisphere.

Figure S9. Forest plots of effect sizes comparing parcel-wise cortical thickness between the adolescence-limited group and the low group

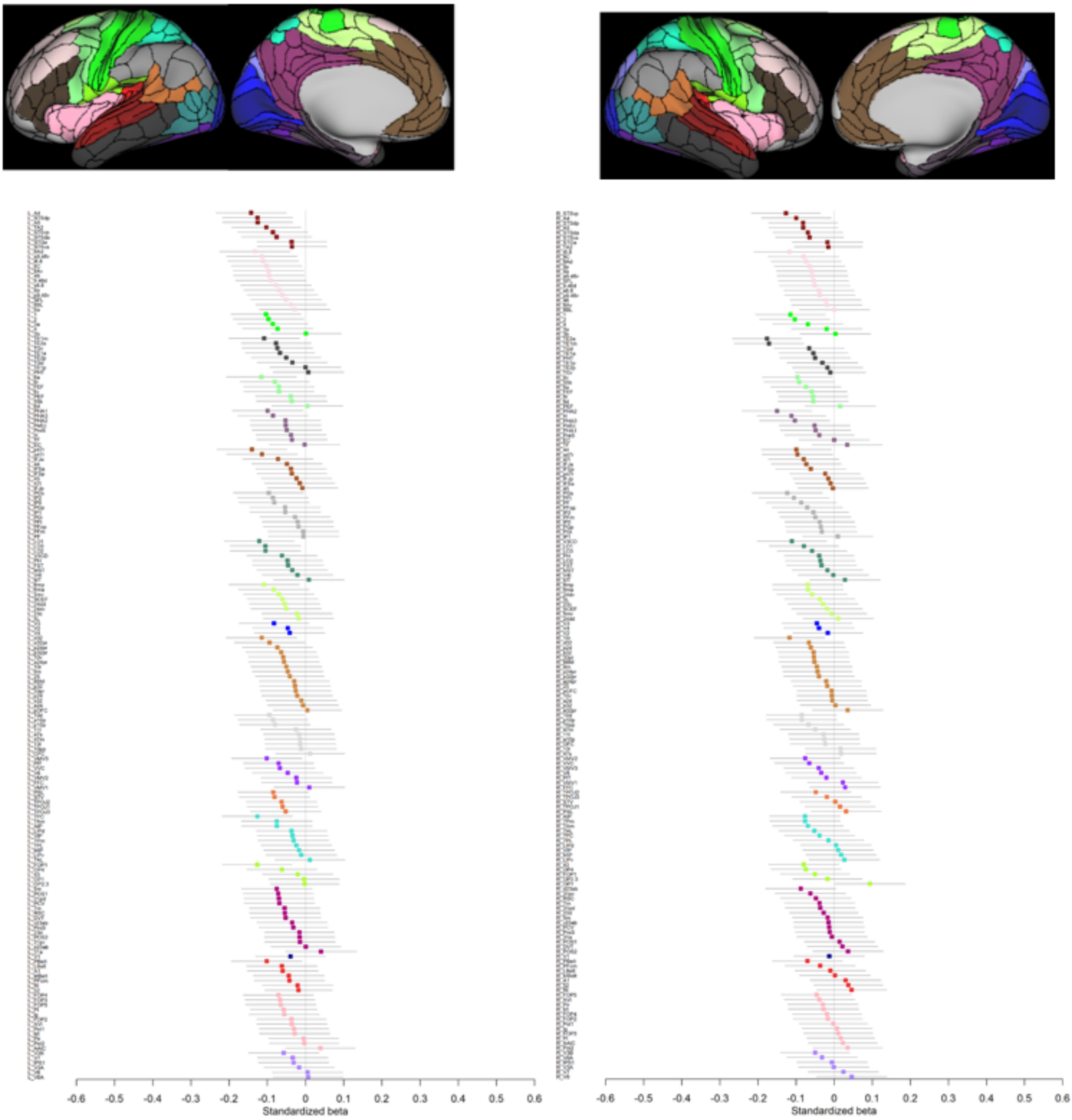

Figure S9. Forest plots of effect sizes comparing parcel-wise cortical thickness between adolescence-limited group and the low group. Forest plots showing parcel-wise effect sizes (represented by standardized beta values) for each parcel in the parcellation scheme denoted by the brain maps in this figure and outlined in Glasser et al., 2016[14]. Each parcel is colour-coded according to 22 broader regions based on anatomical and functional criteria outlined in Glasser et al., 2016[14]. Parcels are then sorted by magnitude of effect size within each region. The left side of the figure corresponds with the left hemisphere.

Figure S10. Forest plots of effect sizes comparing parcel-wise cortical thickness between the life-course-persistent and adolescent-limited groups

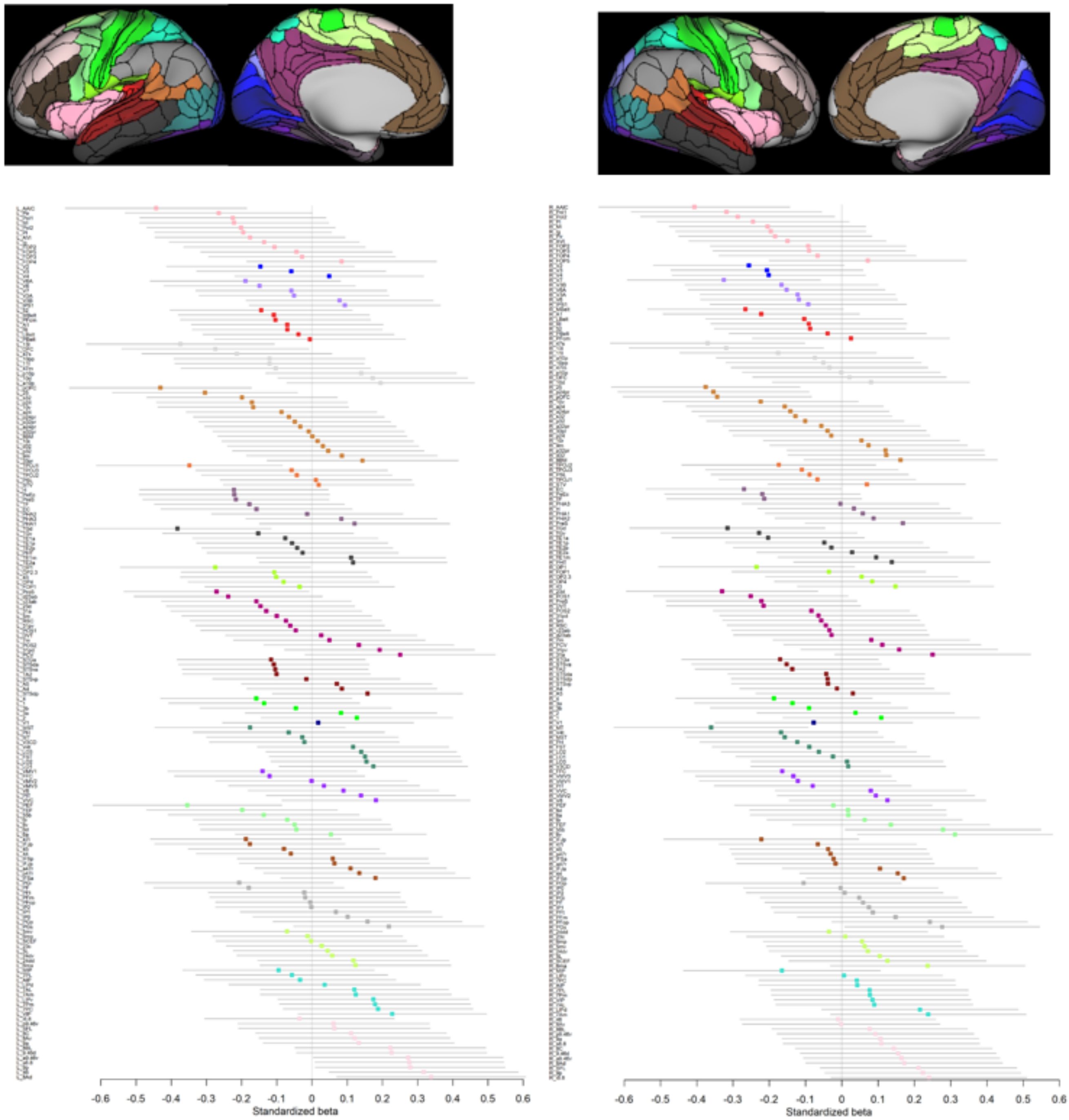

Figure S10. Forest plots of effect sizes comparing parcel-wise cortical thickness between the life-course-persistent and adolescence-limited groups. Forest plots showing parcel-wise effect sizes (represented by standardized beta values) for each parcel in the parcellation scheme denoted by the brain maps in this figure and outlined in Glasser et al., 2016[14]. Each parcel is colour-coded according to 22 broader regions based on anatomical and functional criteria outlined in Glasser et al., 2016[14]. Parcels are then sorted by magnitude of effect size within each region. The left side of the figure corresponds with the left hemisphere.

ADDITIONAL EXPLORATORY ANALYSES AND RESULTS

Confirmation of global findings

To confirm the robustness and reliability of the main global findings reported in the main text Table 2, we repeated these analyses using permutation tests. Results of these tests are reported in **Table S2** below. All original findings were robust to permutation tests.

Table S2. Global surface area and mean average cortical thickness and group comparisons

	Global surface area	*Average cortical thickness*
**Life-course-persistent**<br>Mean (SD)	181204.72 (17382.35)	2.54 (0.10)
**Adolescence-limited**<br>Mean (SD)	186208.29 (16809.61)	2.55 (0.09)
**Low**<br>Mean (SD)	187000.77 (16135.46)	2.56 (0.08)
**Life-course-persistent vs. low**	standardized β=-0.18<br>95% CI: -0.24 - -0.11<br>p<0.0001	standardized β=-0.10<br>95% CI: -0.19 - -0.02<br>p=0.020
p (permutation)	p<0.0001	p=0.0036
**Adolescence-limited vs. low**	standardized β=-0.06<br>95% CI: -0.12 - 0.00<br>p=0.071	standardized β=-0.08<br>95% CI: -0.16 - 0.00<br>p=0.039
p (permutation)	p=0.064	p=0.037
**Life-course-persistent vs. adolescence-limited**	standardized β=-0.17<br>95% CI: -0.26 - -0.07<br>p=0.00078	standardized β=-0.04<br>95% CI: -0.17 - 0.09<br>p=0.56
p (permutation)	p<0.0001	p=0.33

Parcel-wise effects using Family-Wise Error (FWE) correction

To confirm that our findings were not prone to false positives when comparing differences between groups, we repeated our parcel-wise analyses using the more conservative family-wise error (FWE) correction with a threshold of $p<0.05$ (**Figures S11 & S12**). While some parcels which were significant using a false discovery rate procedure did not survive FWE correction, results led to similar overall inference with regard to the observed pattern of findings. That is, the life-course-persistent individuals still showed greater differences when compared to those without antisocial behaviour than did the adolescent-limited individuals. Regional findings which did survive FWE correction were in comparable regions to those observed when FDR correction was used.

Figure S11. Differences in parcel-wise surface area between antisocial behavior trajectory groups and the low antisocial behavior group

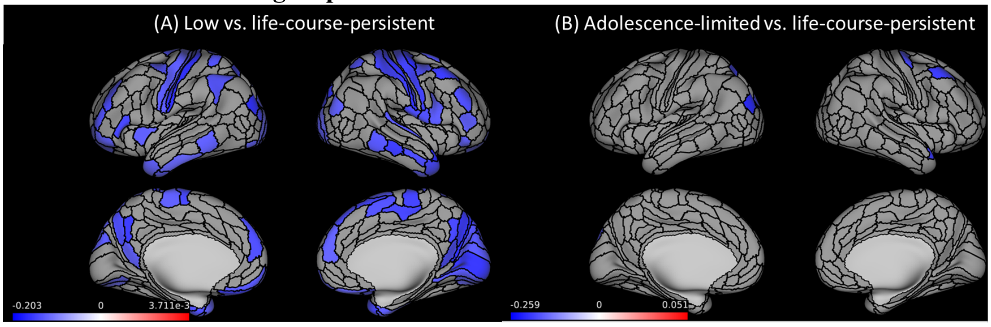

Figure S11. Differences in parcel-wise surface area between antisocial behavior trajectory groups and the low antisocial behavior group. Panel (A) shows parcel-wise regions (in blue) with significantly smaller surface area in Study members with life-course-persistent antisocial behavior compared to Study members in the low group who never exhibited high levels of antisocial behavior. Panel (B) shows parcel-wise regions (in blue) with significantly smaller surface area in Study members with life-course-persistent antisocial behavior compared to those with adolescence-limited antisocial behavior. Not shown is the comparison between the adolescence-limited antisocial group and the low group, as no significant parcels were observed. All results are controlled for sex and family-wise error (FWE) corrected, $p<0.05$.

Figure S12. Differences in parcel-wise cortical thickness between the life-course-persistent antisocial behavior trajectory group and the low antisocial behavior group

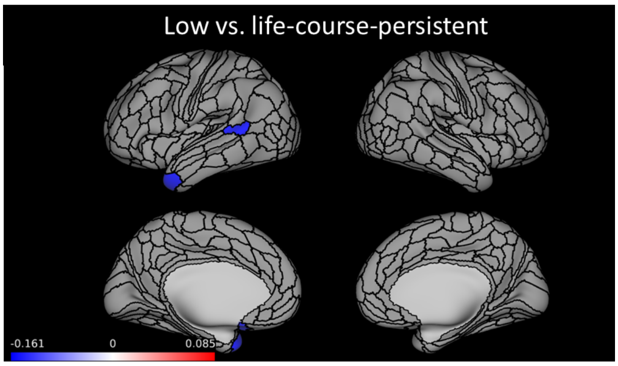

Figure S12. Differences in parcel-wise cortical thickness between the life-course-persistent antisocial behavior trajectory group and the low antisocial behavior group. Images show parcel-wise regions (in blue) with significantly thinner cortex in Study members with life-course-persistent antisocial behavior. Not shown is the comparison between the adolescence-limited group and the low group, or the life-course-persistent and adolescence-limited antisocial groups, as no significant parcels were observed. All results are controlled for sex and family-wise error (FWE) corrected, $p<0.05$.

For a comprehensive approach, our analyses corrected for 360 tests for each of the 360 parcels in our parcellation scheme. However, the number of independent signals is not directly related to the number of parcels (which are not completely independent). To more accurately estimate the effective number of tests being conducted in parcel-wise analyses, we conducted exploratory analyses using the 'meff' function in R.[15] This method estimates the effective number of tests from the correlations among the variables being tested and showed that the effective number of tests to correct for is 279.2 for surface area and 339.7 for cortical thickness, suggesting that even the findings using a FWE procedure for 360 tests may still be overly conservative.

Exploratory analyses of covariates

To examine the potential confounding effects of factors such as total intracranial volume, childhood SES and IQ, history of head injury, and diagnosis of schizophrenia, we conducted a series of exploratory analyses on global measures of surface area and cortical thickness. The results of these analyses are presented below in **Tables S3 and S4**. Controlling for these factors did not drastically change the inference of the original findings, and effect sizes were largely similar, regardless of statistical significance.

Table S3. Exploratory analyses of group comparisons of global surface area

	Global surface area	*Control total intracranial volume*	*Control childhood IQ*	*Control childhood SES*	*Control head injury history*	*Exclude schizophrenia*
**Life-course-persistent vs. low**	standardized β=-0.18<br>95% CI: -0.24 - -0.11<br>p<0.0001	standardized β=-0.05<br>95% CI: -0.08 - -0.02<br>p=0.002	standardized β=-0.12<br>95% CI: -0.19 - -0.05<br>p=0.00063	standardized β=-0.16<br>95% CI: -0.23 - -0.09<br>p<0.0001	standardized β=-0.18<br>95% CI: -0.25 - -0.11<br>p<0.0001	standardized β=-0.17<br>95% CI: -0.24 - -0.10<br>p<0.0001
**Adolescence-limited vs. low**	standardized β=-0.06<br>95% CI: -0.12 - 0.00<br>p=0.071	standardized β=0<br>95% CI: -0.03 - 0.03<br>p=0.844	standardized β=-0.04<br>95% CI: -0.11 - 0.02<br>p=0.15	standardized β=-0.04<br>95% CI: -0.10 - 0.03<br>p=0.24	standardized β=-0.06<br>95% CI: -0.12 - 0.01<br>p=0.075	standardized β=-0.06<br>95% CI: -0.13 - 0.00<br>p=0.062
**Life-course-persistent vs. adolescence-limited**	standardized β=-0.17<br>95% CI: -0.26 - -0.07<br>p=0.00078	standardized β= -0.06<br>95% CI: -0.11- -0.02<br>p=0.011	standardized β=-0.10<br>95% CI: -0.19 - 0.00<br>p=0.049	standardized β=-0.14<br>95% CI: -0.24 - -0.05<br>p=0.0038	standardized β=-0.17<br>95% CI: -0.27 - -0.07<br>p=0.00065	standardized β=-0.16<br>95% CI: -0.26 - -0.06<br>p=0.0018

Table S4. Exploratory analyses of group comparisons of average cortical thickness

	Average cortical thickness	*Control total intracranial volume*	*Control childhood IQ*	*Control childhood SES*	*Control head injury history*	*Exclude schizophrenia*
**Life-course-persistent vs. low**	standardized β=-0.10<br>95% CI: -0.19 - -0.02<br>p=0.020	standardized β= -0.08<br>95% CI: -0.17 - 0<br>p=0.064	standardized β=-0.09<br>95% CI: -0.17 - 0.00<br>p=0.062	standardized β=-0.09<br>95% CI: -0.18 - 0.00<br>p=0.041	standardized β=-0.10<br>95% CI: -0.19 - -0.02<br>p=0.021	standardized β=-0.10<br>95% CI: -0.18 - 0.01<br>p=0.031
**Adolescence-limited vs. low**	standardized β=-0.08<br>95% CI: -0.16 - 0.00<br>p=0.039	standardized β=-0.07<br>95% CI: -0.15 - 0.01<br>p=0.077	standardized β=-0.07<br>95% CI: -0.15 - 0.01<br>p=0.073	standardized β=-0.07<br>95% CI: -0.15 - 0.01<br>p=0.10	standardized β=-0.08<br>95% CI: -0.16 - 0.00<br>p=0.052	standardized β=-0.07<br>95% CI: -0.15 - 0.01<br>p=0.079
**Life-course-persistent vs. adolescence-limited**	standardized β=-0.04<br>95% CI: -0.17 - 0.09<br>p=0.56	standardized β= -0.02<br>95% CI: -0.15 - 0.11<br>p=0.811	standardized β=0.00<br>95% CI: -0.13 - 0.14<br>p=0.95	standardized β=-0.02<br>95% CI: -0.15 - 0.12<br>p=0.80	standardized β=-0.0<br>95% CI: -0.17 - 0.09<br>p=0.53	standardized β=-0.05<br>95% CI: -0.18 - 0.08<br>p=0.47

We also repeated parcel-wise analyses controlling for total surface area and average cortical thickness to examine relative effects. In every group comparison, all 360 parcels were significantly different between groups (FDR corrected, p<0.05), following the same general pattern as in the parcel-wise analyses that did not control for global measures.

References

1 Elley W, Irving J. Revised socio-economic index for New Zealand. *New Zealand Journal of Educational Studies* 1976; **11**: 25–36.

2 Lazarević LB, Knežević G, Mitić M, *et al.* Wechsler Adult Intelligence Scale — Fourth Edition (WAIS – IV). 2015. DOI:10.2298/psi1710010011.

3 Romer AL, Knodt AR, Sison ML, *et al.* Reproducibility of Structural Brain Alterations Associated with Transdiagnostic Risk for Mental Illness: Evidence from a Population-Representative Birth Cohort. *bioRxiv* 2019; : 667220.

4 Lahey BB, Applegate B, Hakes JK, Zald DH, Hariri AR, Rathouz PJ. Is there a general factor of prevalent psychopathology during adulthood? *Journal of abnormal psychology* 2012; **121**: 971.

5 Caspi A, Houts RM, Belsky DW, *et al.* The p Factor: One General Psychopathology Factor in the Structure of Psychiatric Disorders? *Clinical psychological science : a journal of the Association for Psychological Science* 2014; **2**: 119–37.

6 Caspi A, Houts RM, Belsky DW, *et al.* Childhood forecasting of a small segment of the population with large economic burden. *Nature Human Behaviour* 2017; **1**: 0005.

7 Wechsler D. Wechsler Intelligence Scale for Children - Revised, 5th edn. New York: The Psychological Corporation, 1974.

8 Odgers CL, Caspi A, Broadbent JM, *et al.* Prediction of Differential Adult Health Burden by Conduct Problem Subtypes in Males. *Arch Gen Psychiatry* 2007; **64**: 476–84.

9 Odgers CL, Milne BJ, Caspi A, Crump R, Poulton R, Moffitt TE. Predicting Prognosis for the Conduct-Problem Boy: Can Family History Help? *Journal of the American Academy of Child & Adolescent Psychiatry* 2007; **46**: 1240–9.

10 Odgers CL, Moffitt TE, Broadbent JM, *et al.* Female and male antisocial trajectories: From childhood origins to adult outcomes. *Development and Psychopathology* 2008; **20**: 673–716.

11 Moffitt TE. Adolescence-limited and life-course-persistent antisocial behavior: A developmental taxonomy. *Psychological Review* 1993; **100**: 674–701.

12 Greve DN, Fischl B. Accurate and robust brain image alignment using boundary-based registration. *NeuroImage* 2009; **48**: 63–72.

13 Robinson EC, Jbabdi S, Glasser MF, *et al.* MSM: A new flexible framework for Multimodal Surface Matching. *NeuroImage* 2014; **100**: 414–26.

14 Glasser MF, Coalson TS, Robinson EC, *et al.* A multi-modal parcellation of human cerebral cortex. *Nature* 2016; **536**: 171–8.

15 Derringer J. A simple correction for non-independent tests. *Psyarxiv* 2018; published online April 16. DOI:10.31234/osf.io/f2tyw.
